# To Investigate Yellow Fever: The Following Is Promulgated

**Published:** 1897-11

**Authors:** 


### To Investigate Yellow Fever: The Following is Promulgated:

AMERICAN PUBLIC HEALTH ASSOCIATION,
OFFICE OF THE SECRETARY,
COLUMBUS, OHIO, November 2nd, 1897.

At a meeting of the American Public Health Association, held in Philadelphia, October 26–29, 1897, the following resolution was adopted:

*Resolved*, That a committee of seven be appointed to wait on the President of the United States and lay before him the urgent necessity, as viewed by the American Public Health Association, for the appointment by Congress of a Commission of expert bacteriologists, to be sent to Havana, for the purpose of making a thorough study of the cause and prevention of yellow fever.

The following committee was appointed in accordance with the above resolution: Dr. H. S. Holbrook, Charleston, S. C.; Dr. S. H. Burgin, Boston, Mass.; Dr. A. H. Doty, New York, N. Y; Dr. R. M. Swearingen, Austin, Texas; Dr. G. M. Sternberg, U. S. A.; Dr. Josiah Hartzell, Canton, Ohio; Dr. S. R. Olliphant, New Orleans, La.

Yours truly,

C. C. PROBST, Secretary.

This is a move in the right direction. Yellow fever has been "investigated" frequently, but it will bear a good deal more investigation.

